# Identification of a novel compound targeting the nuclear export of influenza A virus nucleoprotein

**DOI:** 10.1111/jcmm.13467

**Published:** 2017-11-30

**Authors:** Feng Huang, Jingliang Chen, Junsong Zhang, Likai Tan, Gui Lu, Yongjie Luo, Ting Pan, Juanran Liang, Qianwen Li, Baohong Luo, Hui Zhang, Gen Lu

**Affiliations:** ^1^ Department of Respiration Affiliated Guangzhou Women and Children's Hospital Zhongshan School of Medicine Sun Yat‐Sen University Guangzhou China; ^2^ Institute of Human Virology Zhongshan School of Medicine Sun Yat‐sen University Guangzhou China; ^3^ Key Laboratory of Tropical Disease Control of Ministry of Education Zhongshan School of Medicine Sun Yat‐sen University Guangzhou China; ^4^ Guangdong Engineering Research Center for Antimicrobial Agent and Immunotechnology Zhongshan School of Medicine Sun Yat‐sen University Guangzhou China; ^5^ Institute of Medicinal Chemistry School of Pharmaceutical Sciences Sun Yat‐sen University Guangzhou China

**Keywords:** influenza A virus, compound ZBMD‐1, nucleoprotein, nuclear export

## Abstract

Although antiviral drugs are available for the treatment of influenza infection, it is an urgent requirement to develop new antiviral drugs regarding the emergence of drug‐resistant viruses. The nucleoprotein (NP) is conserved among all influenza A viruses (IAVs) and has no cellular equivalent. Therefore, NP is an ideal target for the development of new IAV inhibitors. In this study, we identified a novel anti‐influenza compound, ZBMD‐1, from a library of 20,000 compounds using cell‐based influenza A infection assays. We found that ZBMD‐1 inhibited the replication of H1N1 and H3N2 influenza A virus strains *in vitro*, with an IC_50_ ranging from 0.41–1.14 μM. Furthermore, ZBMD‐1 inhibited the polymerase activity and specifically impaired the nuclear export of NP. Further investigation indicated that ZBMD‐1 binds to the nuclear export signal 3 (NES3) domain and the dimer interface of the NP pocket. ZBMD‐1 also protected mice that were challenged with lethal doses of A/PR/8/1934 (H1N1) virus, effectively relieving lung histopathology changes, as well as strongly inhibiting the expression of pro‐inflammatory cytokines/chemokines, without inducing toxicity effects in mice. These results suggest that ZBMD‐1 is a promising anti‐influenza compound which can be further investigated as a useful strategy against IAVs in the future.

## Introduction

Influenza A virus (IAV) is an important human viral pathogen that is responsible for periodic human pandemics as well as seasonal influenza, resulting in substantial human morbidity and mortality and a worldwide financial burden annually 1, 2, 3. Vaccines are currently available to control infections in humans. However, mutations in the haemagglutinin (HA) and neuraminidase (NA) proteins of circulating viruses easily escape the surveillance by the host immune system 4. Specific antiviral drugs are available for prophylaxis and therapeutic treatment for individuals infected with IAV. Two classes of Food and Drug Administration (FDA)‐approved anti‐influenza drugs are currently used in the treatment of IAV infections including NA inhibitors such as oseltamivir and zanamivir 5, and matrix protein 2 (M2) ion channel inhibitors such as amantadine and rimantadine 6, 7. However, resistance development is a serious problem for antiviral drugs, particularly when the target viral proteins continuously undergo a high frequency of antigenic drift 8, 9, 10. Most human influenza viruses, including pandemic 2009 H1N1 and H7N9, are currently resistant to amantadine/rimantadine or oseltamivir 11, 12, 13, 14. Therefore, identification of novel antiviral targets and development of antiviral drugs for the treatment of influenza virus infections are imperative.

The viral nucleoprotein (NP) of influenza A virus has recently been identified as a target for development of antiviral drugs 15, 16, 17, 18, 19. Influenza NP is the major component of the viral ribonucleoprotein (vRNP) complex and abundantly expressed during infection with multiple functions 20. The vRNP complex utilizes the nuclear localization signals (NLSs) within NP for nuclear import through the cellular importin‐α/β‐dependent nuclear import pathway 21, which is also supported by our previous report 22. During the late stage of infection, NP, nuclear export protein (NEP) and matrix protein (M1) mediate the transport of the newly assembled vRNP complexes from the nucleus to the cytoplasm. 21. Overall, NP has several conformations and activities that are conserved among all influenza A viruses and has no cellular equivalent 15. Thus, NP is an ideal target for the development of commercial anti‐influenza drugs.

This study aimed to identify new anti‐influenza compounds by high‐throughput screening of a library consisting of 20,000 compounds using a cell‐based infection assay. We identified compound ZBMD‐1, which inhibited IAV replication and showed low cell toxicity *in vitro* and *in vivo*. It was shown that ZBMD‐1 inhibits the viral polymerase activity of influenza viruses and the nuclear export of NP. Furthermore, docking analysis, surface plasmon resonance (SPR) analysis and co‐immunoprecipitation assay showed that ZBMD‐1 binds to a small pocket structure on the NP molecule.

## Materials and methods

### Screening for compound

#### Compounds

The chemical library of 20,000 structurally diverse small molecule compounds (Chemdiv) was purchased from J&K Chemical Company (Shanghai, China). ZBDM‐1 (≥95% purity) was synthesized by Institute of Medicinal Chemistry (SYSU) and stocked in dimethyl sulphoxide (DMSO). Oseltamivir phosphate (Os) was purchased from Selleck Company (Shanghai, China), and a stock solution was prepared in DMSO. Leptomycin B (LMB) was purchased from Cell Signaling Technology (Beverly, MA, USA) and dissolved in ethanol (EtOH). Lipopolysaccharides (LPS) were purchased from Sigma‐Aldrich (St. Louis, MO, USA), and a stock solution was prepared in water.

#### Cells and viruses

Human lung carcinoma A549 cells, human embryonic kidney 293T cells and the Madin–Darby canine kidney (MDCK) cells were maintained in Dulbecco's modified Eagle's medium (DMEM; Gibco, Los Angeles, CA, USA) supplemented with 10% foetal bovine serum (FBS; Gibco), and 1% penicillin–streptomycin at 37°C with 5% CO_2_.

Influenza A/PR/8/34 (H1N1) virus, A/Guangdong/1/2009 (H1N1) virus and A/Aichi/2/68 (H3N2) virus were used. The A/Aichi/2/68 (H3N2) virus was purchased from American Type Culture Collection (ATCC, VR‐1680). Virus stock was prepared as previously described 22.

#### Plasmids and antibodies

The eight fragments of influenza A/PR/8/34 (H1N1) virus and the vRNP gene fragments of A/Anhui/1/2013 (H7N9) virus were synthesized by Invitrogen. All fragments from both H1N1pdm virus and A/PR/8/34 viruses were cloned into pHW2000 vector separately as previously described 22.

The C‐terminal HA‐tagged or FLAG‐tagged NP‐expressing plasmids, and mutant NP‐expressing plasmids were derived from influenza A/PR/8/34 virus and cloned into pcDNA3.1 vector separately. The antibodies used in our report included anti‐HA (mouse monoclonal, MBL, Japan), anti‐FLAG (rabbit polyclonal, MBL), anti‐GAPDH (rabbit polyclonal, Proteintech Group, Chicago, IL, USA), anti‐NP (rabbit polyclonal, Abcam, Cambridge, MA, USA), anti‐Lamin B1 (rabbit polyclonal, Proteintech Group), anti‐CRM1 (mouse monoclonal, BD Biosciences, San Jose, CA, USA) and anti‐M1 antibodies (rabbit polyclonal, Sino biological, China).

#### Generation of recombinant influenza A viruses

The influenza A/PR/8/34 (H1N1) virus and A/Guangdong/1/2009 (H1N1) virus were rescued using an eight plasmid‐based reverse genetic system as described previously 22.

#### MDCK cell‐based influenza A infection assays

Madin–Darby canine kidney (MDCK) cells were seeded onto 96‐well plates and incubated with DMEM containing a synthesized library compounds using a Tecan Freedom EVO150 (Tecan, Männedorf, Schweiz) at a final concentration of 50 μM. Column 12 received only DMSO instead of any of the compounds. The cells were further infected with influenza A/PR/8/34 virus (H1N1) at a multiplicity of infection (MOI) of 0.005. At 48 hrs post‐infection (p.i.), the CellTiter 96 AQueous One Solution Reagent and an electron coupling reagent were added to each well as recommended by the manufacturer. After culturing for 2 hrs, the cell viability was determined by measuring the absorbance at a wavelength of 490 nm. The screening assay was performed twice in the study.

### *In vitro* testing

#### Plaque assay of antiviral activity

Anti‐influenza A virus activity of ZBMD‐1 was tested according to the previous reports 15. The virus titres were determined by plaque assay on MDCK cells as previously described 22. Briefly, cells were infected with influenza A/PR/8/34 (H1N1) virus at an MOI of 0.001. At 1 hr p.i., the medium was replaced with DMEM containing 0.5% BSA and 1 μg/ml of TPCK‐trypsin. Supernatants were collected at 48 hrs p.i. for measuring virus titres. The virus titres were determined by plaque assay on MDCK cells. MDCK cells were seeded in 12‐well plates and used for infection when the cells were grown to 100% confluence. The MDCK cells were washed with phosphate‐buffered saline (PBS) once and infected with a series of dilutions of viruses for 1 hr at 37°C with 5% CO_2_. After the virus inocula were removed, the cells were washed with PBS, and then overlaid with agarose medium (DMEM containing 0.6% BSA, 2 μg/ml of TPCK‐trypsin, and 1% low‐melting‐point agarose [Sigma‐Aldrich]). The plates were settled at 4°C for 5–10 min. until the agarose medium became solid, followed by culture upside down at 37°C, and then the cells were cultured for 48–72 hrs. Visible plaques were counted, and the 50% inhibitory concentration was determined by counting the number of plaques.

#### Cell toxicity test

Cell toxicity assay was determined by MTS assay according to the manufacturer's instructions (Promega, Madison, WI) as previously described 23. The data are shown as means ±standard deviation (S.D.) from three independent experiments.

#### Influenza A virus minigenome system for polymerase activity

Influenza A virus minigenome system was performed as previously described 22. The data are shown as means ± S.D. from three independent experiments.

#### Western blotting

Cells were lysed with ice‐cold lysis buffer for 30 min. at 4°C. The lysates were then collected and separated by SDS‐PAGE as previously described 22. The bands were immunoblotted with the indicated primary antibodies and IRDye secondary antibodies (LI‐COR, Lincoln, NE, USA), and visualized on an Odyssey infrared imaging system (LI‐COR). The relative protein expression level was analysed using the software provided in the Odyssey system.

#### Co‐immunoprecipitation assay

Human 293T cells were seeded at 6‐cm dishes and transfected with various plasmids as indicated. Cells were then untreated or treated with different amount of ZBMD‐1 at 12 hrs p.t.. At 48 hrs p.t., cells were lysed with 450 μl of ice‐cold lysis buffer. About 10% (40 μl) of the lysates was taken as input control. The remaining lysates were incubated with anti‐HA agarose bead for 4 hrs at 4°C. The beads were then washed three times with 500 μl of ice‐cold lysis buffer, followed by Western blotting.

#### Immunofluorescence assay (IFAs)

Immunofluorescence assay (IFAs) were performed as previously described 22, 24, 25. Briefly, 293T cells were fixed with 4% paraformaldehyde, permeabilized with 1% Triton X‐100, subsequently blocked with 5% BSA blocking solution and stained with primary antibodies and secondary antibodies. Cell nuclei were stained with 4′, 6‐diamidino‐2‐phenylindole (DAPI) reagent (Invitrogen, Carlsbad, CA, USA). Images were obtained by a Leica laser scanning microscope using the Leica software (Wetzlar, Germany).

#### Nuclear and cytoplasmic protein fractionation

Human 293T cells transfected with NP‐HA‐expressing plasmid were untreated or treated with ZBMD‐1. Cells were harvested and washed with PBS at 48 hrs p.t. Fractionation of cytoplasmic and nuclear components was performed according to the manufacturer's instructions (PARIS, Millipore, MA, USA) as previously described 22, 26.

#### The expression of GFP analysed with FACS

The cells transfected with AID‐GFP with different treatment were collected and fixed with formaldehyde and then analysed with the BD LSR Fortessa™ cell analyzer according to the manufacturer's protocol (BD) as previously described 24, 27.

### *In silico* docking model

#### Molecular docking analysis

The crystal structure of influenza A virus nucleoprotein was downloaded from RCSB Protein Data Bank (PDB code: 2IQH, resolution: 3.2 Å). ZBMD‐1 and NP binding was assessed using DOCK 6.7 28, 29. The ligand and receptor structures were constructed using UCSF Chimera 30. The DOCK6 program was then utilized to conduct semi‐flexible docking where 1000 different orientations were generated. Van der Waals and electrostatic interactions were obtained between the ligand and the binding site, which were then used in calculating the Grid scores. We used a molecular visualization tool PyMOL 31 (The PyMOL Molecular Graphics System, Version 1.8, Schrödinger, Seattle, USA) to generate the surfaces of NP to help spotting the binding sites of ZBMD‐1 on the interfaces of NP.

#### Surface plasmon resonance assay

The assays were performed using a Biacore T100 instrument (GE Healthcare, Amersham, UK) as previously described with some modification 23. A Biacore CM5 Sensor Chip and an amine coupling kit were purchased from GE Healthcare. The optimal pH for NP‐His immobilization (pH 4.0) was first determined. The CM5 censor chip was activated and then injected with NP‐His (300 mg/ml in 10 mM acetate buffer, pH 4.0) for 7 min. The residual activated groups on the surfaces were blocked by injecting ethanolamine HCl (1 M) for 7 min. Different amounts of ZBMD‐1 were diluted with 1% DMSO and then injected for 30 min.

### *In vivo* testing

#### Ethics statement

All animal experiments were approved by Ethics Committee of Zhongshan School of Medicine (ZSSOM) on Laboratory Animal Care and were carried out in strict accordance with the guidelines and regulations of Laboratory Animal Center of ZSSOM, SYSU, Guangzhou (China) (Assurance Number: 2016‐053). Mice in the study were provided *via* standard pellet feed and water. All procedures were performed under anaesthesia that was induced by isoflurane (RWD Life Science Co., Ltd., Shenzhen, China) treatment, and all efforts were made to minimize suffering. Mice were killed if rapid and lasting weight loss (loss of more than 25% of body weight in a few days) was observed.

#### *In vivo* antiviral activity assay

ZBMD‐1 was dissolved in DMSO and then diluted with corn oil (Sigma‐Aldrich) to a final concentration of 2 mg/ml. DMSO diluted with corn oil was taken as control. Male 6‐week‐old BALB/c mice (approximately 20 g) were used in our experiments. Mice were intranasally infected with ten 50% lethal doses (LD_50_) of A/PR/8/34 (H1N1) and then intraperitoneally injected with ZBMD‐1 twice daily (5, 10, or 20 mg/kg) for 5 days beginning at 6 hrs after infection, respectively. In the co‐administration experiment, mice were intraperitoneally co‐administered or given monotherapy with 10 mg/kg of ZBMD‐1 and 0.2 mg/kg of oseltamivir phosphate twice daily for 5 days beginning at 6 hrs after infection, respectively. Eight mice per treatment group were tested. Body weight and survival rates of each group were measured daily. In parallel experiments, five mice in each group were killed on day 3 p.i., and their lungs were removed for determination of viral titres using the plaque assay. All experiments were repeated in triplicate. Differences in body weight or lung viral titre between each group were compared from control using one‐way anova analysis, while survival rates were analysed by the log‐rank test. Data were considered significant at *P <* *0.05, **P <* *0.01 and ***P <* *0.001.

### Quantitative real‐time PCR

Total RNA isolation and qRT‐PCR were performed as previously described 26. Specific primers for the target genes TNF‐α, IL‐1β, IL‐6, CCL‐2, CXCL1, IL‐6 and GAPDH (as housekeeping control) of mice were designed using Primer 5.0 based on the corresponding gene sequences of the proteins. The primer sequences were as follows: IL‐6‐F: 5′‐CCCCAATTTCCAATGCTCTCC‐3′; IL‐6‐R: 5′‐CGCACTAGGTTTGCCGAGTA‐3′; IL‐1β‐F: 5′‐TCGCTCAGGGTCACAAGAA‐3′; IL‐1β‐R: 5′‐GTGCTGCCTAATGTCCCCTT‐3′; TNFα‐F: 5′‐ATGGCTCAGGGTCCAACTCT‐3′; TNFα‐R: 5′‐CGAGGCTCCAGTGAATTCGG‐3′; CCL2‐F: 5′‐AACTGCATCTGCCCTAAGGT‐3′; CCL2‐R: 5′‐AGGCATCACAGTCCGAGTCA‐3′; CXCL1‐F: 5′‐ACTCAAGAATGGTCGCGAGG‐3′; CXCL1‐R: 5′‐GTGCCATCAGAGCAGTCTGT‐3′; HA‐F: 5′‐TATTTGGAGCCATTGCCGGT‐3′; HA‐R: 5′‐GATCCGCTGCATAGCCTGAT‐3′. The data are shown as means ± S.D. from three independent experiments.

### Histopathological analysis

Half of the lung (or liver, spleen, kidney, heart) of each mouse was fixed in formalin, embedded in paraffin and then stained with haematoxylin and eosin (H&E) for histological analysis. Slides were randomized, read blindly and examined for tissue damage and inflammatory cellular infiltration. The lung pathology was scored in a range of 0–4 as previous report 32.

### Statistical analysis

Data were analysed using GraphPad Prism 6.0 software (La Jolla, CA, USA). The two‐tailed Student's *t*‐test, one‐way anova and the log‐rank test were used to determine the significance of statistical data. Data were considered significant at *P <* *0.05, **P <* *0.01 and ***P < 0.001.

## Results

### Screening for compound

**Figure 1:**
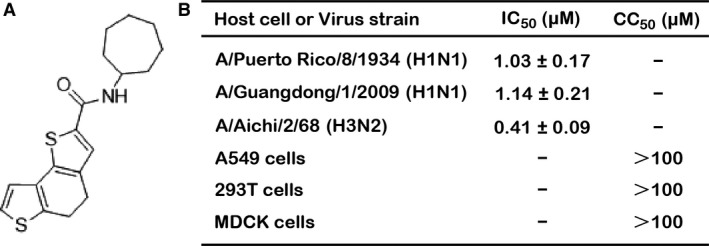
Effect of compound ZBMD‐1 on the replication of influenza A virus. (**A**) The chemical structure of compound ZBMD‐1 is shown. (**B**) ZBMD‐1 is effective against human H1N1 and H3N2 influenza viruses. MDCK cells were infected with different strains of the virus at an MOI of 0.001 in the presence of different doses of ZBMD‐1. The 50% inhibitory concentration (IC_50_) of ZBMD‐1 against the A/PR/8/34 (H1N1) virus was 1.03 μM, against A/Guangdong/1/2009 (H1N1) virus was 1.14 μM and against A/Aichi/2/68 (H3N2) virus was 0.41 μM in a plaque assay. The 50% cytotoxicity concentration (CC_50_) was measured using an MTS assay.

To discover novel anti‐influenza compounds, an MDCK cell‐based influenza A infection high‐throughput screening was performed. Briefly, a chemical library of 20,000 structurally diverse small molecule compounds (50 μM) was applied in our screening after MDCK cells infected with A/PR/8/34 (H1N1) virus (multiplicity of infection [MOI] = 0.005 PFU). We then performed a dose–response assay among the hit compounds and identified seven compounds that exerted an inhibitory effect by more than 50% at a concentration of 10 μM. Among the anti‐influenza hits, compound ZBMD‐1 showed the highest potency against influenza A virus in a plaque reduction assay (PRA) on MDCK cells infected with A/PR/8/34 (H1N1) virus. The chemical structure of compound ZBMD‐1 was shown in Figure 1A. Using a plaque assay, the 50% inhibitory concentration (IC_50_) of ZBMD‐1 against A/PR/8/34 (H1N1) in MDCK cells was lower than 2 μM (Fig. 1B). ZBMD‐1 showed a similar level of antiviral activity against different IAV subtypes including A/Guangdong/1/2009 (H1N1) virus (IC_50_ = 1.14 ± 0.21 μM) and A/Aichi/2/68 (H3N2) virus (IC_50_ = 0.41 ± 0.09 μM) (Fig. 1B). Furthermore, ZBMD‐1 had low toxicity in MDCK, A549 and 293T cells. The 50% cytotoxic concentration (CC_50_) of the compound was >100 μM (Fig. 1B). Together, these results show that compound ZBMD‐1 significantly inhibits the replication of IAVs.

### *In vitro* testing

**Figure 2:**
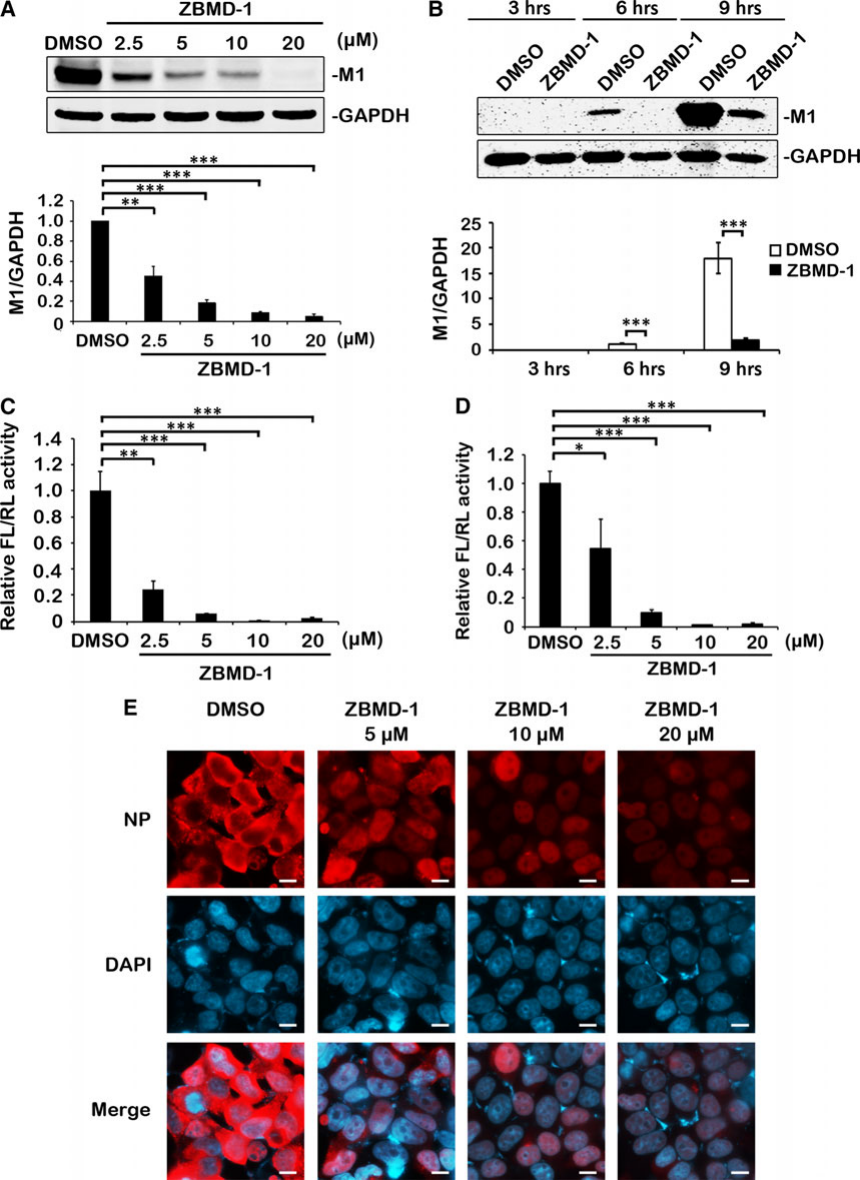
Effect of ZBMD‐1 on viral polymerase activity. (**A**) ZBMD‐1 inhibits the protein synthesis of influenza A viruses (IAV) in a dose‐dependent manner. A549 cells treated with different amounts of ZBMD‐1 were infected with A/PR/8/34 virus at an MOI of 1 and collected at 12 hrs after infection. The cells were lysed for Western blotting (top panel). Bottom panel, statistical analysis of M1 levels in the top panel. The value for M1 was standardized to that of the GAPDH levels and normalized to the level of M1 in cells transfected with DMSO. (**B**) ZBMD‐1 inhibits the protein synthesis of IAV in a time‐course manner. A549 cells treated with 10 μM of ZBMD‐1 were infected with A/PR/8/34 (H1N1) virus at an MOI of 1 and collected at different time‐points after infection (top panel). The cells were lysed for Western blotting analysis. Bottom panel, statistical analysis of M1 levels in the top panel. The value for M1 was standardized to that of the GAPDH levels and normalized to the level of M1 in cells transfected with DMSO at 3 hrs. (**C**) ZBMD‐1 impairs polymerase activity of A/PR/8/34 (H1N1) virus in a dose‐dependent manner in the minigenome system. Human 293T cells were transfected in triplicate with plasmids for the minigenome system. Increasing amounts of ZBMD‐1 were added onto the cells at 12 hrs p.t. The cells were collected at 48 hrs p.t. and were then used in a dual‐luciferase reporter assay. (**D**) The effect of ZBMD‐1 on the polymerase activity of the A/Anhui/1/2013 (H7N9) virus in the minigenome system. Human 293T cells were transfected in triplicate with plasmids for the minigenome system. Increasing amounts of ZBMD‐1 were added to the cells at 12 hrs p.t.. The cells were collected at 48 hrs p.t. and used in a dual‐luciferase reporter assay. Data are shown as the means ± S.D. from three independent experiments. Differences between each group from DMSO were tested using Student's *t*‐test: *, *P *<* *0.05; **, *P *<* *0.01; ***, *P *<* *0.001. (**E**) ZBMD‐1 affects the nucleocytoplasmic distribution of vRNP in infected cells. Human A549 cells infected with the A/PR/8/34 virus at an MOI of 5 were treated with different amounts of ZBMD‐1 at 4 hrs p.i. At 12 hrs p.i., the cells were fixed and immunostained for NP (red) and nuclei (blue). Scale bars, 10 μm. Each group was scored using random fields of view with triplicates.

To further confirm the inhibitory effect of ZBMD‐1 on the replication of IAV, we examined the effect of ZBMD‐1 on viral protein synthesis. We observed a significant dose‐dependent decrease in viral protein synthesis after treatment with ZBMD‐1 compared with the control (dimethyl sulphoxide; DMSO) (Fig. 2A). Furthermore, the Western blot analysis showed that the levels of viral M1 protein were decreased compared with the control as early as 6 hrs p.i. (Fig. 2B), further implying that ZBMD‐1 inhibits the replication of influenza A virus.

To explore the target of ZBMD‐1, we explored the inhibitory activity of ZBMD‐1 through two additional assays: the pseudotyped virus expressing influenza haemagglutinin (HA) 33 and the dual‐luciferase minigenome system 34. We found that ZBMD‐1 did not inhibit the entry of IAV in the pseudotyped virus reporter system (Data not shown). The dual‐luciferase minigenome system showed that ZBMD‐1 significantly inhibited viral RNA polymerase activity in a dose‐dependent manner (Fig. 2C). ZBMD‐1 also effectively decreased the polymerase activity of the influenza H7N9 strain (Fig. 2D). These results indicate that ZBMD‐1 inhibits the activity of vRNP complex.

In our previous study, we demonstrated that blocking vRNP nuclear transport impairs the activity of vRNP complex 22. To further confirm the inhibitory effect of ZBMD‐1 on the viral polymerase activity of infected cells, we performed an immunofluorescence assay (IFA) to examine whether ZBMD‐1 affects the nuclear transport of vRNP. Because influenza virus NP is the major component of the vRNP complex, the location of NP in infected cells represents the location of vRNP complex 1, 21. Accordingly, a reduction in viral NP protein expression was also detected by IFA assay (Fig. 2E). We found that NP in the cells treated with DMSO (negative control) was predominantly located in the cytoplasm at 12 hrs p.i.. However, after treated with ZBMD‐1, most of NP was located in the nucleus (Fig. 2E), indicating that in the presence of ZBMD‐1, the vRNP complex was able to enter the nucleus but was not exported to the cytoplasm afterwards. The retention of NP in the nucleus was more obvious with higher doses of ZBMD‐1. These results show that ZBMD‐1 affects the nucleocytoplasmic distribution of influenza virus vRNP complex during infection.

**Figure 3:**
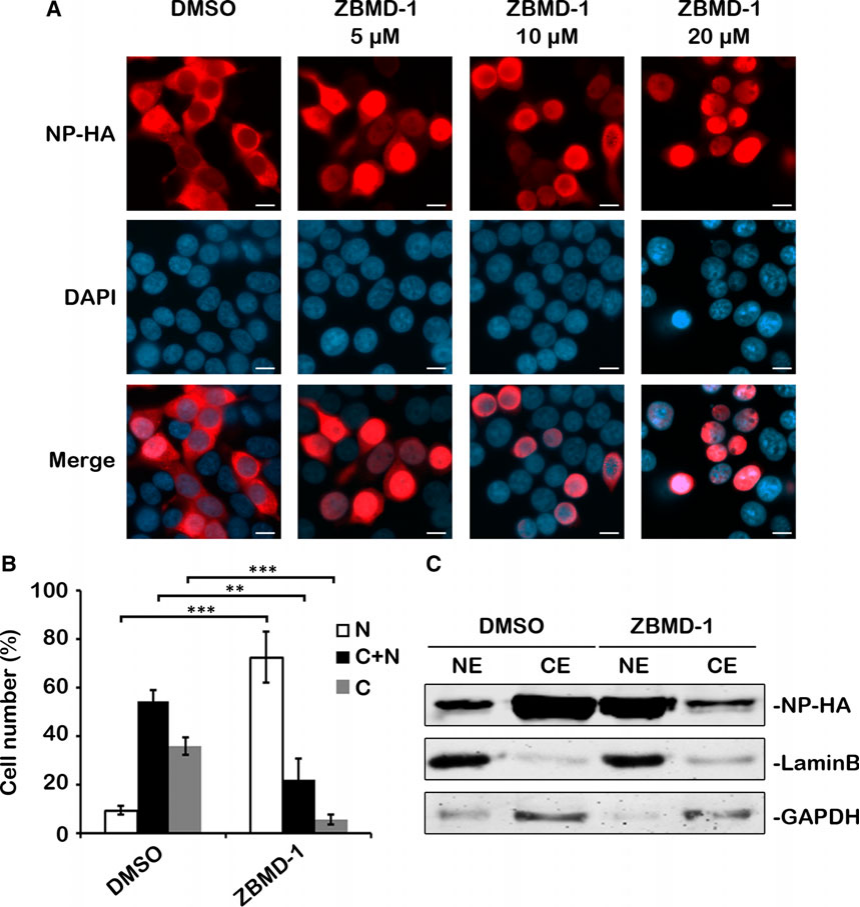
ZBMD‐1 affects the nucleocytoplasmic distribution of nucleoprotein (NP). (**A**) Human 293T cells transfected with NP‐HA‐expressing plasmid were treated with different amounts of ZBMD‐1 and fixed at 24 hrs p.t., followed by immunofluorescence using anti‐HA antibody (red). The nucleus was stained with DAPI (blue). Scale bars, 10 μm. (**B**) Quantitative analysis of the nucleocytoplasmic distribution of NP. At least 200 cells in the 10 μM group from three independent assays were scored. N, predominantly nuclear; N; C, nuclear and cytoplasmic; C, predominantly cytoplasmic. Data were expressed as the mean ± S.D. of three independent experiments. Differences between each group from DMSO were tested using Student's *t*‐test: *, *P *<* *0.05; **, *P *<* *0.01; ***, *P *<* *0.001. (**C**) The cells transfected with NP‐HA and treated with ZBMD‐1 (DMSO as control) were separated into cytoplasmic (C) and nuclear (N) fractions. Each fraction was examined by Western blotting.

The influenza virus NP is a nuclear shuttle protein that contains both nuclear localization signals (NLSs) and nuclear export signals (NESs) 20. Among the vRNP complex components, only NP has a known NES 1. Therefore, the retention of vRNP complex in the nucleus might due to a disruption in the nuclear export of NP. To elucidate our hypothesis, we investigated the effects of ZBMD‐1 on the nuclear export of NP by IFA. Indeed, we found that NP was mainly detected in the cytoplasm at 24 hrs post‐transfection (p.t.) in the absence of ZBMD‐1, but was mostly retained in the nucleus with ZBMD‐1, and the cytoplasmic distribution of NP was dependent on the dose of ZBMD‐1 (Fig. 3A). The subcellular localization of NP in the presence of 10 μM of compounds in each group was scored using random fields of view with triplicates. The results demonstrated that most NP was located in the nucleus in the presence of ZBMD‐1 (Fig. 3B). Accordingly, when we repeated the assay using nuclear–cytoplasmic fractionation, a lower cytoplasmic ratio of NP was observed with ZBMD‐1 treatment (Fig. 3C).

**Figure 4:**
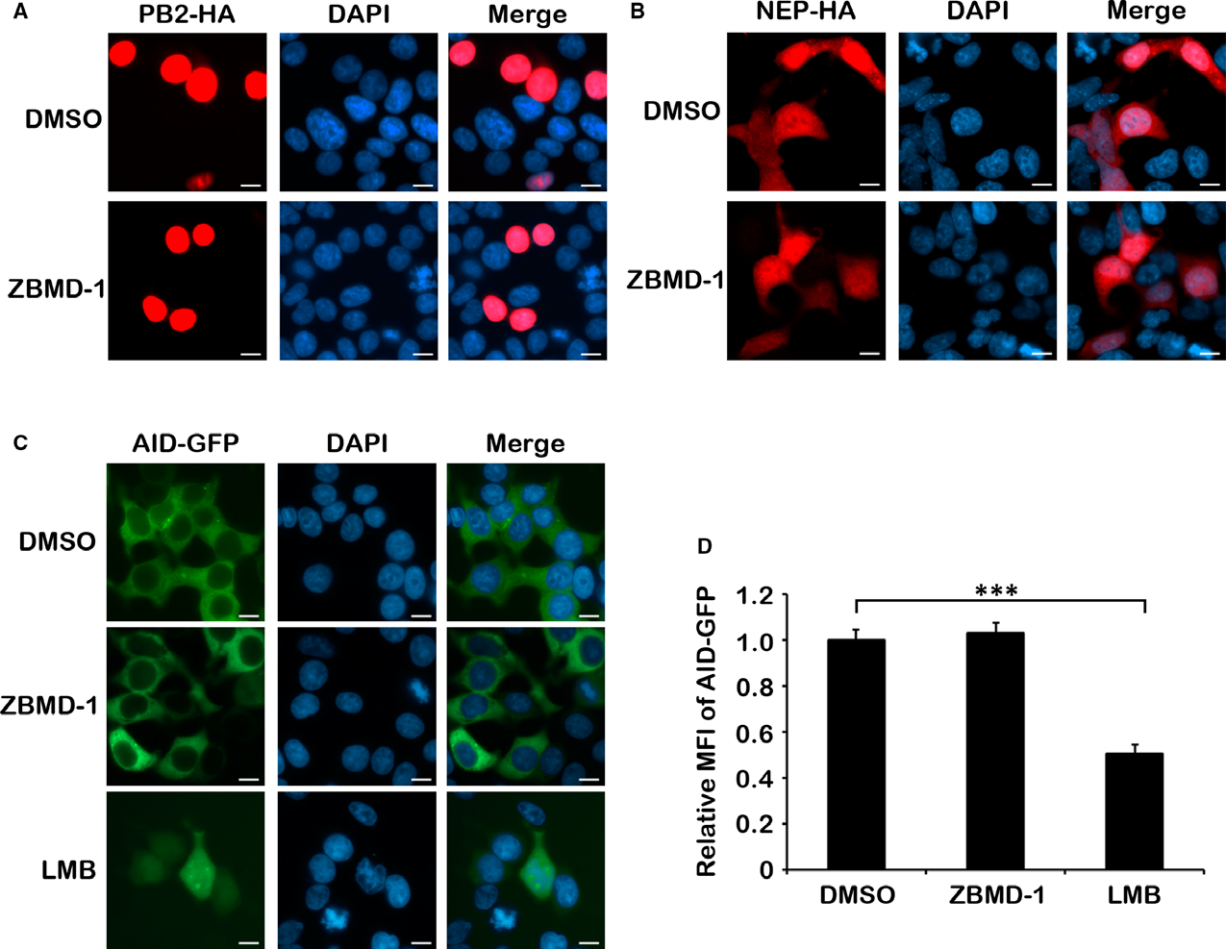
ZBMD‐1 specifically inhibits the nuclear export of nucleoprotein (NP). **(A** and **B)** ZBMD‐1 has no effect on the distribution of PB2 or NEP in cells. Human 293T cells transfected with a PB2‐HA‐expressing plasmid (**A**) or NEP‐HA‐expressing plasmid (**B**) were treated with DMSO or ZBMD‐1 (20 μM) at 12 hrs p.t. and then fixed at 24 hrs p.t., which were then followed by immunofluorescence using anti‐HA antibody (red). (**C**) The effect of ZBMD‐1 on the nuclear export of AID. Human 293T cells transfected with AID‐GFP‐expressing plasmid were treated with DMSO or ZBMD‐1 (20 μM) at 12 hrs p.t. and then fixed at 24 hrs p.t., which was then followed by fluorescence microscopy analysis. Scale bars, 10 μm. Each group was scored using random fields of view with triplicates. (**D**) ZBMD‐1 does not affect AID‐GFP nuclear stability. Human 293T cells transfected with AID‐GFP‐expressing plasmids were treated with DMSO, ZBMD‐1 (20 μM) or LMB (10 nM) at 12 hrs p.t. and then fixed at 24 hrs p.t., which were then followed by FACS analysis. Data are shown as the means ± S.D. from three independent experiments. ***, *P *<* *0.001 (Student's *t*‐test).

However, the localization of PB2, another major protein of vRNP complex 1, was not altered with ZBMD‐1 treatment (Fig. 4A). Among the vRNP complex proteins, only NP has a known NES; however, IAV in the absence of NEP cannot export NP from the nucleus, therefore indicating that NEP is necessary for the nuclear export of vRNP complex 21. Thus, we further analysed the location of NEP after ZBMD‐1 treatment, and found that the nuclear location of NEP was not affected by ZBMD‐1 treatment (Fig. 4B), indicating that the effect of ZBMD‐1 is specific for the nuclear export of NP.

IAVs adapt the cellular CRM1‐dependent nuclear export pathway to transport vRNP complex from the nucleus to the cytoplasm 35, 36. Nucleoprotein (NP) is retained in the nucleus in the presence of a specific CRM1 inhibitor, leptomycin B (LMB) 37. To exclude the possibility that ZBMD‐1 is an LMB‐like CRM1 inhibitor, we examined the effect of ZBMD‐1 on the nuclear export of activation‐induced deaminase (AID), a well‐known host protein that is exported from the nucleus *via* the CRM‐1 nuclear export pathway 38. Figure 4C shows that ZBMD‐1 did not inhibit the nuclear export of AID‐GFP, whereas LMB treatment did. AID‐GFP is unstable in the nucleus 39, and LMB treatment resulted in the degradation of AID‐GFP (Fig. 4D). However, ZBMD‐1 treatment did not affect the expression of AID‐GFP. Together, these results suggest that ZBMD‐1 specifically targets on NP, but not on the CRM1 transport system.

#### *In silico* docking model

**Figure 5:**
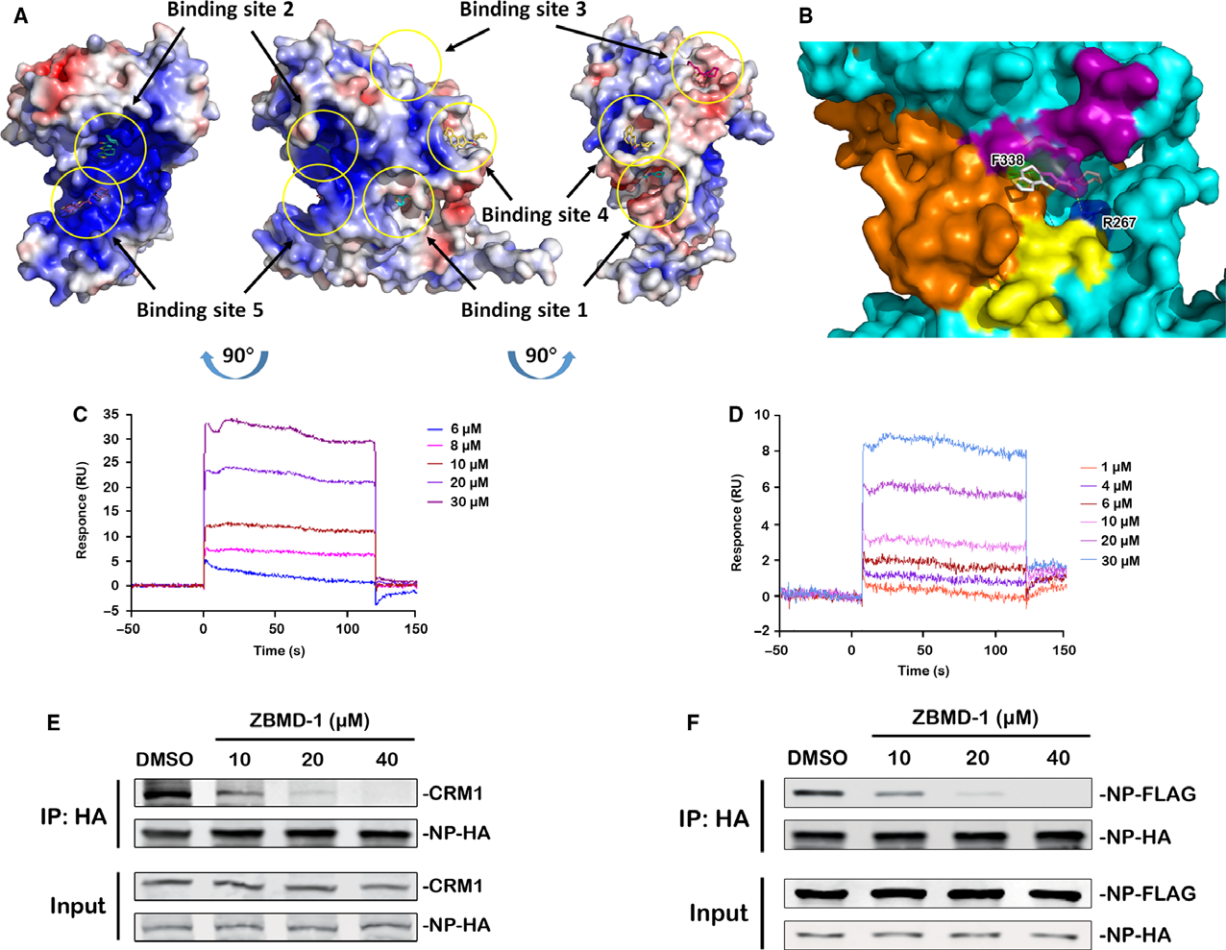
Influenza nucleoprotein (NP) is the molecular target of ZBMD‐1. (**A**) The potential binding sites (highlighted by yellow circles) of ZBMD‐1 on the influenza A NP crystal structure as predicted by in silico docking analysis. Red, negative charge; blue, positive charge; light grey, neutral charge. (**B**) Close‐up view of the indicated small pocket. Two predictive binding residues (R267 and F338) within NP are shown. The nuclear export signal 3 (NES) domain (amino acid [aa] 256–266) is shown as yellow; RNA binding grove (aa 1–180) is shown as orange; NP dimer interface (aa 482–489) is shown as purple; the binding pocket of ZBMD‐1 within NP surrounding F338 is shown as green, and pocket surrounding R267 is blue. (**C** and **D**) The binding of ZBMD‐1 to influenza NP (**C**) or its mutant (**D**) was evaluated using a SPR assay. The sensorgrams were obtained by injecting a series of concentrations of ZBMD‐1 over the immobilized NP or mutant NP chip. BIA evaluation software was used to determine the equilibrium dissociation constant (Kd). The binding affinity of ZBMD‐1 with wild‐type NP‐His or mutant NP‐His with Kd values of 10.6 (**C**) or 131.6 μmol/l (**D**), respectively. Two independent experiments were performed and one representative result is shown, respectively. (**E**) The effect of ZBMD‐1 on the interaction between NP and CRM1. Human 293T cells were transfected with NP‐HA‐expressing plasmid and treated with different amounts of ZBMD‐1 at 12 hrs p.t. The cells were collected and lysed for immunoprecipitation using anti‐HA agarose. The associated CRM1 was determined by Western blotting with an anti‐CRM1 antibody. (**F**) Effect of ZBMD‐1 on NP‐NP interaction. Human 293T cells were transfected with NP‐HA‐ and NP‐FLAG‐expressing plasmids and treated with different amounts of ZBMD‐1 at 12 hrs p.t. The cells were collected and lysed for immunoprecipitation with anti‐HA agarose. The associated NP‐FLAG was determined by Western blotting using an anti‐FLAG antibody.

To further confirm whether NP is a direct molecular target of ZBMD‐1, we utilized the published crystal structure of influenza A/WSN/33 (H1N1) NP from the Protein Data Bank (PDB) for *in silico* docking studies. For unbiased predictive docking analysis, we searched for potential docking sites within the entire NP monomer. Five potential binding sites were identified (Fig. 5A). Based on the highest binding free energy, binding site 1 of ZBMD‐1 within NP was selected for further analysis. The configuration with the best binding score (ΔG of −48.37 kcal/mol) was also identified and used in further studies (Fig. 5B). Two amino acid residues (F338 and R267, hydrogen bond) play a major role in ZBMD‐1 binding to NP (Fig. 5B). To further confirm the accuracy of this binding model, a surface plasmon resonance (SPR) experiment was utilized to examine whether ZBMD‐1 binds to wild‐type and mutant NP proteins harbouring alanine substitutions within potential binding sites (from R267 to 267A and F338 to 338A mutant). The SPR assay indicated that ZBMD‐1 dose‐dependently bound to the wild‐type NP‐His or mutant NP‐His with Kd values of 10.6 or 131.6 μmol/l, respectively (Fig. 5C and D). Although ZBMD‐1 remained bind to His‐tagged mutant NP protein, its binding affinity was significantly lower than that to wild‐type NP protein, thereby indicating that these two residues play critical roles in the interaction between ZBMD‐1 and NP. These results suggest that ZBMD‐1 binds to NP mainly through amino acid residues, R267 and F338, which were predicted by *in silico* docking analysis.

ZBMD‐1 binds to the NES3 region of NP (aa 248‐274), which is involved in the nuclear export of NP 40, thereby indicating the mechanism underlying the nuclear export of NP. A previous study has shown that the nuclear export mediated by NES1 or NES2 is CRM1‐independent, whereas that by NES3 is CRM1‐dependent 40. To further investigate whether the inhibitory effect of ZBMD‐1 on the nuclear export of NP depends on the CRM1 nuclear transport pathway, we examined the binding of endogenous CRM1 to NP in the presence or absence of ZBMD‐1. The binding between NP and CRM1 was inhibited by ZBMD‐1 in a dose‐dependent manner (Fig. 5E). This result indicates that ZBMD‐1 inhibits the binding between NP and CRM1 through interacting with NES domain of NP.

In addition to the NP amino acids involved in NP‐CRM1 interactions, the docking model showed that ZBMD‐1 partially bound to amino acids involving the NP dimer domain 41. To examine whether ZBMD‐1 induces the dissociation of NP‐NP homo‐oligomer, we performed a co‐IP assay incorporating NP proteins that was fused to different tags as previous report 18. In the absence of ZBMD‐1, NP‐FLAG was able to bind to the HA agarose beads that were coupled with NP‐HA (Fig. 5F). However, ZBMD‐1 inhibited the binding of NP‐FLAG to NP‐HA in a dose‐dependent manner, suggesting that ZBMD‐1 also inhibits the NP‐NP interaction. Taken together, these results demonstrate that ZBMD‐1 inhibits both the function of NES3 domain and oligomerization of NP, but not the NP‐RNA interaction.

#### *In vivo* testing

**Figure 6:**
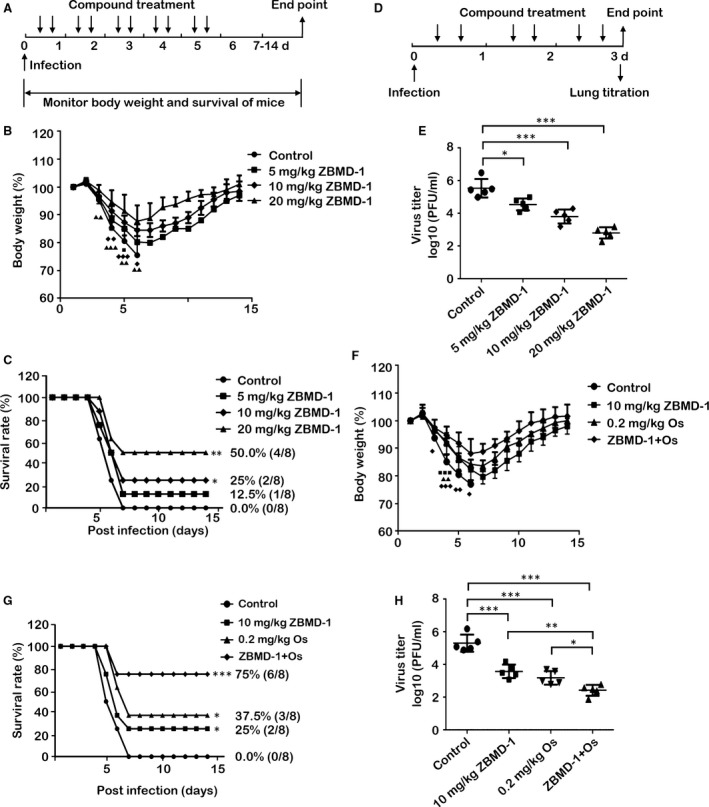
The effect of ZBMD‐1 on IAV replication *in vivo*. (**A‐C**) Mice were infected intranasally with 10 LD_50_ of influenza A/PR/8/34 (H1N1) virus. Different amounts of ZBMD‐1 were intraperitoneally injected into 6‐week‐old BALB/c mice at 6 hrs after virus exposure and twice per day for 5 days beginning on the day of infection. DMSO diluted in corn oil was used as negative control. Eight mice per treatment group were tested. The body weight of mice from each group was monitored daily (**B**), and survival rates were also calculated (**C**). Body weight at day 0 was set as 100%. ■, group 5 mg/kg of ZBMD‐1, *P *<* *0.05; **♦**, group 10 mg/kg of ZBMD‐1, *P *<* *0.05; **♦♦**,* P *<* *0.01; **♦♦♦**,* P *<* *0.001; **▲**, 20 mg/kg of ZBMD‐1, *P *<* *0.05; **▲▲**,* P *<* *0.01; **▲▲▲**,* P *<* *0.001 (Student's *t*‐test). The survival rates of each group were compared with control using the log‐rank test. *, *P *<* *0.05; **, *P *<* *0.01. (**D‐E**) Five mice from the each group were killed on day 3 p.i., and the lungs were collected for the determination of viral titres. The data were expressed as the mean ± S.D. (**F‐H**) The antiviral effect of co‐administration of ZBMD‐1 and oseltamivir phosphate *in vivo*. ZBMD‐1 (10 mg/kg) and oseltamivir phosphate (Os, 0.2 mg/kg) were intraperitoneally co‐administered or given monotherapy into 6‐week‐old BALB/c mice twice per day for 5 days beginning on the day of infection, respectively. The body weight of mice from each group was monitored daily (**F**), and survival rates were also calculated (**G**). Body weight at day 0 was set as 100%. ■, group 10 mg/kg of ZBMD‐1, *P *<* *0.05; ■■■, *P *<* *0.001; **▲**, 0.2 mg/kg of Os, *P *<* *0.05; **▲▲**,* P *<* *0.01; **▲▲▲**,* P *<* *0.001; **♦**, group co‐administration of ZBMD‐1 and oseltamivir phosphate, *P *<* *0.05; **♦♦**,* P *<* *0.01; **♦♦♦**,* P *<* *0.001. Body weights of each group were compared with control group using one‐way anova. Survival rates of each group were compared with control using the log‐rank test. *, *P *<* *0.05; **, *P *<* *0.01; ***, *P *<* *0.001. In parallel experiment, lung titre of each group was analysed at day 3 p.i. (**H**). Differences between each group from control were tested using one‐way anova: *, *P *<* *0.05; **, *P *<* *0.01; ***, *P *<* *0.001. All experiments were repeated for three times. Body weight change and survival rate of each group were showed by a single experiment.

We further tested the *in vivo* efficacy of ZBMD‐1 against IAV infection. Mice were inoculated with 10 LD_50_ of A/PR/8/34 (H1N1) and intraperitoneally injected daily with different doses of ZBMD‐1 (5, 10 or 20 mg/kg twice per day) for five days beginning at 6 hrs after infection, respectively (Fig. 6A). ZBMD‐1 treatment apparently protected the mice from influenza‐induced morbidity and lethality (Fig. 6B and C). In a parallel experiment, mice were killed from each group at 3d p.i., and the lungs were isolated and used in the determination of virus titre by plaque assay (Fig. 6D and E). The virus load in lungs of ZBMD‐1‐treated mice was significantly lower than that of control group. Moreover, ZBMD‐1 and oseltamivir phosphate were co‐administrated to investigate the antiviral activity of ZBMD‐1 treated together with oseltamivir phosphate. Although ZBMD‐1 was less effectively protected the mice from influenza *in vivo* compared to that of oseltamivir phosphate, the co‐administration of ZBMD‐1 and oseltamivir phosphate resulted in an even lower degree of weight loss and a higher survival rate (up to 75%) than those treated with ZBMD‐1 or oseltamivir phosphate alone (Fig. 6F and G). These results further support the hypothesis that the mechanisms of ZBMD‐1 and oseltamivir phosphate are highly variable and better *in vivo* effect is observed when administered in combination. Co‐administration of ZBMD‐1 and oseltamivir phosphate also significantly reduced the viral load in lung (Fig. 6H). These results show that ZBMD‐1 effectively protects mice from influenza infection *in vivo*.

**Figure 7:**
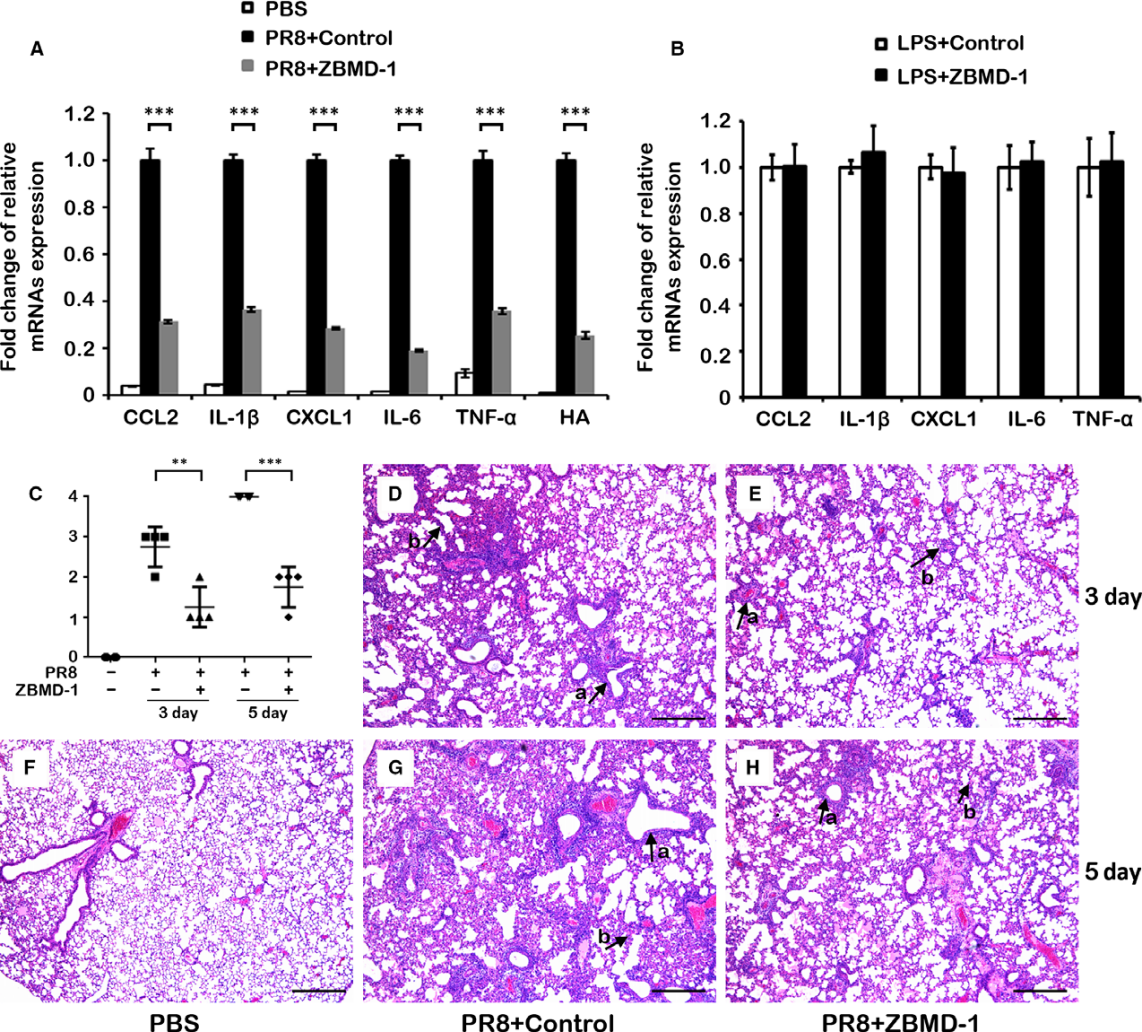
The anti‐inflammatory activity of ZBMD‐1 *in vivo*. (**A**) The relative mRNA expression of cytokines/chemokines was tested. Three mice (at each time‐point) from each group were killed at 3 days p.i., and their lungs were collected for analysis of mRNA expression of pro‐inflammatory cytokines/chemokines, respectively. Influenza HA was taken as positive control in the experiment. (**B**) Mice were injected with 10 mg/kg LPS together with or without ZBMD‐1, and then monitored the relative mRNA expression at 3 days after treatment. The data were expressed as the mean ± S.D. ***, *P *<* *0.001 (Student's *t*‐test). (**C‐H**) ZBMD‐1 effectively attenuated the lung pathology of influenza‐infected mice. Lung samples were subjected to quantitative score of lung pathology (**C**). The data were expressed as the mean ± S.D. **, *P *<* *0.01; ***, *P *<* *0.001 (Student's *t*‐test). Mice from each group (four mice each group) were killed on 3 or 5 days p.i., and lungs were collected, stained with H&E and used in histopathological analysis. Arrow a, infiltration of inflammatory cells; arrow b, alveolar wall thickening. Scale bars, 200 μM.

Influenza virus infection causes severe lung pathology and is associated with acute lung inflammation, which is exacerbated partly through the host immune response to virus infection 42, 43, 44. A down‐regulation of pro‐inflammatory cytokines/chemokines can impede influenza virus‐associated pathogenesis 45. Therefore, we investigated the effect of ZBMD‐1 on influenza virus‐induced pro‐inflammatory cytokine/chemokine expression *in vivo*. Mice were mock infected or infected with 10 LD_50_ of A/PR/8/33(H1N1) virus, and then untreated or treated with ZBMD‐1 (20 mg/kg twice daily). Mice were killed at 3 days p.i., and lungs of the mice were collected for total RNA extraction. The mRNA levels of cytokines that are involved in promoting inflammation and reported to play an important role during severe IAV infections in lungs 46, 47, 48 significantly decreased in ZBMD‐1‐treated mice compared with untreated mice (Fig. 7A). However, treated ZBMD‐1 in mice with lipopolysaccharides (LPS) activation did not alter the expression of TNF‐α, IL‐1β, IL‐6, CXCL1 and CCL2 in lung, indicted that ZBMD‐1 has no effect on the expression of pro‐inflammatory cytokines in the absence of infection (Fig. 7B). These results show that ZBMD‐1 reduces the expression of pro‐inflammatory cytokines that are associated with influenza virus infection.

In a parallel experiment, lungs were collected at 3 or 5 days p.i., and stained with H&E for histological evaluation. ZBMD‐1 treatment alleviated the inflammation and interstitial epithelium thickening that was induced by the IAV infection (Fig. 7C‐H). These observations imply that ZBMD‐1 probably limits further spread of the virus in the lungs, thereby alleviating inflammation. In line with Figure 7, these results also indicate that ZBMD‐1 inhibits the replication of IAV *in vivo*, which in turn alleviates the inflammatory levels and pathology induced by IAV. Our overall findings suggest that ZBMD‐1 has potent anti‐inflammatory effects against the host intense immune response induced by IAV.

## Discussion

In the present study, we identified a novel compound, ZBMD‐1, which efficiently inhibits the replication of influenza A virus *in vitro* and *in vivo*. ZBMD‐1 efficiently protected cells against different subtypes of influenza A virus. In particular, ZBMD‐1 was able to inhibit the replication of A/Aichi/2/68 (H3N2) virus with a relatively low concentration (IC_50_ = 0.41 ± 0.09 nM) *in vitro*. Furthermore, *in vivo* data indicated that ZBMD‐1 reduces the expression of pro‐inflammatory cytokines/chemokines and alleviates the infiltration of inflammation in lungs that is caused by IAV infection. It also increased the survival rate of mice and prevented body weight loss due to IAV infection. These results suggest that ZBMD‐1 effectively inhibits IAV replication and reduces the viral titres *in vitro* and *in vivo*, which in turn further alleviates the pathology induced by IAV. These findings suggest that ZBMD‐1 has potent anti‐inflammatory effects against the host intense immune response induced by IAV, thereby rendering it as a promising lead compound for the treatment of severe influenza.

The *in vivo* co‐administration of ZBMD‐1 and oseltamivir phosphate augments the antiviral effect, suggesting that that ZBMD‐1 inhibited viral replication *via* a new mechanism that differs from that of oseltamivir phosphate. Further investigation indicated that ZBMD‐1 blocks the nuclear transport of vRNP complex from the nucleus through targeting the nuclear export of NP. Three NESs were identified within the NP. Export *via* NES1 and NES2 is independent on the CRM1 transport pathway, whereas export *via* NES3 is dependent on CRM1 binding 40. Therefore, the nuclear retention of NP that was induced by ZBDM‐1 may be due to the disruption of the following pathways: (*i*) ZBMD‐1 targets the NES1 or NES2 within NP; (*ii*) ZBMD‐1 targets the NES3 within NP that further blocks the interaction between NP and CRM1; and (*iii*) ZBMD‐1 targets the CRM1 or its associated proteins. To exclude that CRM1 is the direct target of ZBMD‐1, we utilized an AID‐GFP‐expressing plasmid in our experiments because AID is a classic host protein that is transported from the nucleus and is mainly mediated by the CRM1 pathway 21. The distribution of AID‐GFP was not affected by ZBMD‐1, whereas the classical small molecule inhibitor LMB blocked the nuclear export of AID. Therefore, ZBMD‐1 did not directly inhibit the CRM1 transport pathway as LMB did. To further explore the particular binding sites of ZBMD‐1 within NP, docking analysis that incorporated the structure of ZBMD‐1 within NP was performed. This result showed that the potential binding site of ZBMD‐1 within NP is a small pocket that included NES3. In line with the binding model, further investigation proved that ZBMD‐1 directly binds to NP and disrupts the interaction between NP and CRM1. Moreover, the docking analysis and SPR assay indicated that aa F338 of NP was another important binding site for ZBMD‐1. In line with these results, ZBMD‐1 inhibited the NP‐NP interaction, which further reduced the polymerase activity and impaired the protein synthesis of IAV. Taken together, these results strongly support our theory that NP is the target of ZBMD‐1.

Drug resistance is a serious problem that has been associated with influenza treatment and is caused by high antigenic drift and shift rates. The discovery of new target has thus been the focus on the development of new anti‐influenza therapeutics. Sequence analysis has indicated that NP is a well‐conserved protein among different strains of IAV 15. Furthermore, the target sites of ZBMD‐1 within the NP (R267 and F338) were highly conserved (>99% conservation) 15, thereby indicating that ZBMD‐1 has a broadly inhibitory potency against various IAV strains, including the replication of H1N1 and H3N2 subtypes and the polymerase activity of H7N9 subtype. An alternative challenge for drug design and development is to avoid the occurrence of serious side effects. Influenza NP protein plays a crucial role in the viral life cycle. The activity, assembly, stability and nuclear transport of vRNP complex all rely on the function of NP 1. Therefore, NP is a highly conserved and functionally restricted protein, thus rendering it a promising specific target for anti‐influenza drug design.

Nucleoprotein (NP) has recently considered as a novel target for anti‐influenza drug design 15, 16, 17, 18, 19. Recently, a small compound, RK424, targeting a functional domain within NP has been identified 15. The pocket targeted by this compound was reported to be surrounded by three different domains, namely the RNA binding groove, the NP dimer interface and NES3. In the present study, the binding pocket of ZBMD‐1 within NP in our study was similar to that of RK424. Based on similar function and different chemical structures, we concluded that the binding pocket of ZBMD‐1 is an indispensable pocket of NP, which would be an ideal antiviral target sides. In our studies, ZBMD‐1 was shown to inhibit IAV replication *in vitro* and *in vivo* efficiently. Because ZBMD‐1 is one of the first‐generation hit compounds identified *via* high‐throughput screening, it could be further optimized to become more potent inhibitors.

In summary, a novel compound ZBMD‐1 was identified with a high potency for inhibition of IAV replication *in vitro* and *in vivo*. ZBMD‐1 disrupts the distribution of influenza NP protein in cells, as well as blocks the nuclear export of NP through impeding the binding between NP and CRM1. Meanwhile, the binding of ZBMD‐1 affects NP dimer formation, implying that this compound is a multifunctional NP inhibitor. Thus, the findings of the present study suggest that ZBMD‐1 is a promising anti‐influenza compound which can be further investigated as a useful strategy against IAVs in the future.

## Conflict of interest

The authors confirm that there is no conflict of interests.

## References

[1] Eisfeld AJ , Neumann G , Kawaoka Y . At the centre: influenza A virus ribonucleoproteins. Nat Rev Microbiol. 2015; 13: 28–41. 25417656 10.1038/nrmicro3367 PMC5619696

[2] Molinari NA , Ortega‐Sanchez IR , Messonnier ML , *et al* The annual impact of seasonal influenza in the US: measuring disease burden and costs. Vaccine. 2007; 25: 5086–96. 17544181 10.1016/j.vaccine.2007.03.046

[3] Simonsen L . The global impact of influenza on morbidity and mortality. Vaccine. 1999; 17(suppl 1): S3–10. 10471173 10.1016/s0264-410x(99)00099-7

[4] van der Vries E , Schutten M , Fraaij P , *et al* Influenza virus resistance to antiviral therapy. Adv Pharmacol. 2013; 67: 217–46. 23886002 10.1016/B978-0-12-405880-4.00006-8

[5] Oxford JS , Mann A , Lambkin R . A designer drug against influenza: the NA inhibitor oseltamivir (Tamiflu). Expert Rev Anti Infect Ther. 2003; 1: 337–42. 15482128

[6] Block SL . Role of influenza vaccine for healthy children in the US. Paediatr Drugs. 2004; 6: 199–209. 15339199 10.2165/00148581-200406040-00001

[7] Davies WL , Grunert RR , Haff RF , *et al* Antiviral activity of 1‐Adamantanamine (Amantadine). Science. 1964; 144: 862–3. 14151624 10.1126/science.144.3620.862

[8] Bright RA , Shay DK , Shu B , *et al* Adamantane resistance among influenza A viruses isolated early during the 2005‐2006 influenza season in the United States. JAMA. 2006; 295: 891–4. 16456087 10.1001/jama.295.8.joc60020

[9] Dharan NJ , Gubareva LV , Meyer JJ , *et al* Infections with oseltamivir‐resistant influenza A(H1N1) virus in the United States. JAMA. 2009; 301: 1034–41. 19255110 10.1001/jama.2009.294

[10] Hurt AC , Ernest J , Deng YM , *et al* Emergence and spread of oseltamivir‐resistant A(H1N1) influenza viruses in Oceania, South East Asia and South Africa. Antiviral Res. 2009; 83: 90–3. 19501261 10.1016/j.antiviral.2009.03.003

[11] Chen HL , Cheung CL , Tai H , *et al* Oseltamivir‐resistant influenza A pandemic (H1N1) 2009 virus, Hong Kong, China. Emerg Infect Dis. 2009; 15: 1970–2. 19961677 10.3201/eid1512.091057 PMC3044550

[12] Hauge SH , Dudman S , Borgen K , *et al* Oseltamivir‐resistant influenza viruses A (H1N1), Norway, 2007‐08. Emerg Infect Dis. 2009; 15: 155–62. 19193257 10.3201/eid1502.081031 PMC2657637

[13] Marjuki H , Mishin VP , Chesnokov AP , *et al* Characterization of drug‐resistant influenza A(H7N9) variants isolated from an oseltamivir‐treated patient in Taiwan. J Infect Dis. 2015; 211: 249–57. 25124927 10.1093/infdis/jiu447 PMC6943751

[14] Meijer A , Lackenby A , Hungnes O , *et al* Oseltamivir‐resistant influenza virus A (H1N1), Europe, 2007‐08 season. Emerg Infect Dis. 2009; 15: 552–60. 19331731 10.3201/eid1504.081280 PMC2671453

[15] Kakisaka M , Sasaki Y , Yamada K , *et al* A novel antiviral target structure involved in the RNA binding, dimerization, and nuclear export functions of the influenza A virus nucleoprotein. PLoS Pathog. 2015; 11: e1005062. 26222066 10.1371/journal.ppat.1005062 PMC4519322

[16] Kao RY , Yang D , Lau LS , *et al* Identification of influenza A nucleoprotein as an antiviral target. Nat Biotechnol. 2010; 28: 600–5. 20512121 10.1038/nbt.1638 PMC7097325

[17] Lejal N , Tarus B , Bouguyon E , *et al* Structure‐based discovery of the novel antiviral properties of naproxen against the nucleoprotein of influenza A virus. Antimicrob Agents Chemother. 2013; 57: 2231–42. 23459490 10.1128/AAC.02335-12 PMC3632891

[18] Shen YF , Chen YH , Chu SY , *et al* E339.. R416 salt bridge of nucleoprotein as a feasible target for influenza virus inhibitors. Proc Natl Acad Sci USA. 2011; 108: 16515–20. 21930946 10.1073/pnas.1113107108 PMC3189076

[19] Su CY , Cheng TJ , Lin MI , *et al* High‐throughput identification of compounds targeting influenza RNA‐dependent RNA polymerase activity. Proc Natl Acad Sci USA. 2010; 107: 19151–6. 20974907 10.1073/pnas.1013592107 PMC2984200

[20] Portela A , Digard P . The influenza virus nucleoprotein: a multifunctional RNA‐binding protein pivotal to virus replication. J Gen Virol. 2002; 83: 723–34. 11907320 10.1099/0022-1317-83-4-723

[21] Hutchinson EC , Fodor E . Nuclear import of the influenza A virus transcriptional machinery. Vaccine. 2012; 30: 7353–8. 22652398 10.1016/j.vaccine.2012.04.085

[22] Zhang JS , Huang F , Tan LK , *et al* Host protein moloney leukemia virus 10 (MOV10) Acts as a restriction factor of influenza A virus by inhibiting the nuclear import of the viral nucleoprotein. J Virol. 2016; 90: 3966–80. 26842467 10.1128/JVI.03137-15 PMC4810528

[23] Zhang SY , Zhong LM , Chen B , *et al* Identification of an HIV‐1 replication inhibitor which rescues host restriction factor APOBEC3G in Vif‐APOBEC3G complex. Antiviral Res. 2015; 122: 20–7. 26241003 10.1016/j.antiviral.2015.07.009

[24] Geng GN , Liu BF , Chen CC , *et al* Development of an attenuated tat protein as a highly‐effective agent to specifically activate HIV‐1 latency. Mol Ther. 2016; 24: 1528–37. 27434587 10.1038/mt.2016.117 PMC5113098

[25] Zhou N , Pan T , Zhang JS , *et al* Glycopeptide antibiotics potently inhibit cathepsin L in the Late endosome/lysosome and block the entry of ebola virus, middle east respiratory syndrome coronavirus (MERS‐CoV), and severe acute respiratory syndrome coronavirus (SARS‐CoV). J Biol Chem. 2016; 291: 9218–32. 26953343 10.1074/jbc.M116.716100 PMC4861487

[26] Huang F , Zhang J , Zhang Y , *et al* RNA helicase MOV10 functions as a co‐factor of HIV‐1 Rev to facilitate Rev/RRE‐dependent nuclear export of viral mRNAs. Virology. 2015; 486: 15–26. 26379090 10.1016/j.virol.2015.08.026

[27] Zhong F , Zhou N , Wu K , *et al* A SnoRNA‐derived piRNA interacts with human interleukin‐4 pre‐mRNA and induces its decay in nuclear exosomes. Nucleic Acids Res. 2015; 43: 10474–91. 26405199 10.1093/nar/gkv954 PMC4666397

[28] Lang PT , Brozell SR , Mukherjee S , *et al* DOCK 6: combining techniques to model RNA‐small molecule complexes. RNA. 2009; 15: 1219–30. 19369428 10.1261/rna.1563609 PMC2685511

[29] Mukherjee S , Balius TE , Rizzo RC . Docking validation resources: protein family and ligand flexibility experiments. J Chem Inf Model. 2010; 50: 1986–2000. 21033739 10.1021/ci1001982 PMC3058392

[30] Pettersen EF , Goddard TD , Huang CC , *et al* UCSF chimera ‐ A visualization system for exploratory research and analysis. J Comput Chem. 2004; 25: 1605–12. 15264254 10.1002/jcc.20084

[31] Alexander N , Woetzel N , Meiler J . bcl:cluster: a method for clustering biological molecules coupled with visualization in the pymol molecular graphics system. IEEE Int Conf Comput Adv Bio Med Sci. 2011; 2011: 13–8. 10.1109/ICCABS.2011.5729867 PMC5091839 27818847

[32] Perwitasari O , Johnson S , Yan XZ , *et al* Verdinexor, a novel selective inhibitor of nuclear export, reduces influenza a virus replication *in vitro* and *in vivo* . J Virol. 2014; 88: 10228–43. 24965445 10.1128/JVI.01774-14 PMC4136318

[33] Wang SY , Su CY , Lin MG , *et al* HA‐pseudotyped retroviral vectors for influenza antagonist screening. J Biomol Screen. 2009; 14: 294–302. 19211776 10.1177/1087057108330786

[34] Marklund JK , Ye Q , Dong J , *et al* Sequence in the influenza A virus nucleoprotein required for viral polymerase binding and RNA synthesis. J Virol. 2012; 86: 7292–7. 22532672 10.1128/JVI.00014-12 PMC3416340

[35] Elton D , Simpson‐Holley M , Archer K , *et al* Interaction of the influenza virus nucleoprotein with the cellular CRM1‐mediated nuclear export pathway. J Virol. 2001; 75: 408–19. 11119609 10.1128/JVI.75.1.408-419.2001 PMC113933

[36] Ma K , Roy AMM , Whittaker GR . Nuclear export of influenza virus ribonucleoproteins: identification of an export intermediate at the nuclear periphery. Virology. 2001; 282: 215–20. 11289803 10.1006/viro.2001.0833

[37] Sun QX , Carrasco YP , Hu YC , *et al* Nuclear export inhibition through covalent conjugation and hydrolysis of Leptomycin B by CRM1. Proc Natl Acad Sci USA. 2013; 110: 1303–8. 23297231 10.1073/pnas.1217203110 PMC3557022

[38] Ellyard JI , Benk AS , Taylor B , *et al* The dependence of Ig class‐switching on the nuclear export sequence of AID likely reflects interaction with factors additional to Crm1 exportin. Eur J Immunol. 2011; 41: 485–90. 21268017 10.1002/eji.201041011 PMC3437479

[39] Aoufouchi S , Faili A , Zober C , *et al* Proteasomal degradation restricts the nuclear lifespan of AID. J Exp Med. 2008; 205: 1357–68. 18474627 10.1084/jem.20070950 PMC2413033

[40] Yu MR , Liu XL , Cao S , *et al* Identification and characterization of three novel nuclear export signals in the influenza A virus nucleoprotein. J Virol. 2012; 86: 4970–80. 22345439 10.1128/JVI.06159-11 PMC3347336

[41] Ye Q , Guu TS , Mata DA , *et al* Biochemical and structural evidence in support of a coherent model for the formation of the double‐helical influenza A virus ribonucleoprotein. MBio. 2012; 4: e00467–12. 23269829 10.1128/mBio.00467-12 PMC3531806

[42] Loosli CG . The pathogenesis and pathology of experimental air‐borne influenza virus A infections in mice. J Infect Dis. 1949; 84: 153–68. 18113780 10.1093/infdis/84.2.153

[43] Mauad T , Hajjar LA , Callegari GD , *et al* Lung pathology in fatal novel human influenza A (H1N1) infection. Am J Respir Crit Care Med. 2010; 181: 72–9. 19875682 10.1164/rccm.200909-1420OC

[44] Taubenberger JK , Morens DM . The pathology of influenza virus infections. Annu Rev Pathol. 2008; 3: 499–522. 18039138 10.1146/annurev.pathmechdis.3.121806.154316 PMC2504709

[45] Josset L , Belser JA , Pantin‐Jackwood MJ , *et al* Implication of inflammatory macrophages, nuclear receptors, and interferon regulatory factors in increased virulence of pandemic 2009 H1N1 influenza A virus after host adaptation. J Virol. 2012; 86: 7192–206. 22532695 10.1128/JVI.00563-12 PMC3416346

[46] Ramos I , Fernandez‐Sesma A . Modulating the innate immune response to influenza A virus: potential therapeutic use of anti‐inflammatory drugs. Front Immunol. 2015; 6: 361. 26257731 10.3389/fimmu.2015.00361 PMC4507467

[47] Szretter KJ , Gangappa S , Lu XH , *et al* Role of host cytokine responses in the pathogenesis of avian H5N1 influenza viruses in mice. J Virol. 2007; 81: 2736–44. 17182684 10.1128/JVI.02336-06 PMC1866007

[48] Zheng BJ , Chan KW , Lin YP , *et al* Delayed antiviral plus immunomodulator treatment still reduces mortality in mice infected by high inoculum of influenza A/H5N1 virus. Proc Natl Acad Sci USA. 2008; 105: 8091–6. 18523003 10.1073/pnas.0711942105 PMC2430364

